# New Drugs, Appliances, and Things Medical

**Published:** 1893-03-04

**Authors:** 


### NEW DRUGS, APPLIANCES, AND THINGS MEDICAL.

[All preparations, appliances, novelties, etc., of which a notice is desired, should be sent for the Editor, to care of The Manager, 428, Strand, London, W.C.]

### BRAND AND CO.'S MEAT JUICE.

This well-known firm of invalid food manufacturers have forwarded to us a sample of the above. It is a clear port-coloured fluid of agreeable smell and taste. On heating it becomes almost solid, showing a very large percentage of coagulable albumen. Microscopically and otherwise the fluid gives evidence that it is what it claims to be, a pure extract of beef prepared by pressure in the cold. It is an excellent concentrated food material, and will enhance the reputation of the firm which sends it out. Brand's Meat Lozenges, as well as the Meat Juice form two invaluable aids to the doctor in severe cases of acute illness. Some of both should be found in every household.

### SWINBORNE'S ISINGLASS.

Swinborne's Isinglass is a household word still, though many forms of cornflour have taken its place for thickening milk, not to the advantage of the consumers, especially be they invalids. In fact, Swinborne's Isinglass is quite a necessity in the sick room. Taken with milk, it renders this more digestible, and makes most delicate blanc-mange and jellies. Directions for its preparation are sold with each packet, and it can be obtained at all grocers without difficulty. We do not hesitate to recommend it, as, if once employed, it cannot fail to become a favourite in both household and sick room use.

